# Room-temperature chemical synthesis of C_2_

**DOI:** 10.1038/s41467-020-16025-x

**Published:** 2020-05-01

**Authors:** Kazunori Miyamoto, Shodai Narita, Yui Masumoto, Takahiro Hashishin, Taisei Osawa, Mutsumi Kimura, Masahito Ochiai, Masanobu Uchiyama

**Affiliations:** 1 Graduate School of Pharmaceutical Sciences, The University of Tokyo, 7-3-1 Hongo, Bunkyo-ku, Tokyo, 113-0033 Japan; 2 Division of Chemistry and Materials, Faculty of Textile Science and Technology, Shinshu University, Ueda, 386-8567 Japan; 3 Research Initiative for Supra-Materials (RISM), Shinshu University, Ueda, 386-8567 Japan; 4 Graduate School of Pharmaceutical Sciences, University of Tokushima, 1-78 Shomachi, Tokushima, 770-8505 Japan; 5 Cluster of Pioneering Research (CPR), Advanced Elements Chemistry Laboratory, RIKEN, 2-1 Hirosawa, Wako-shi, Saitama, 351-0198 Japan

**Keywords:** Chemical bonding, Reaction mechanisms, Physical chemistry, Carbon nanotubes and fullerenes

## Abstract

Diatomic carbon (C_2_) is historically an elusive chemical species. It has long been believed that the generation of C_2_ requires extremely high physical energy, such as an electric carbon arc or multiple photon excitation, and so it has been the general consensus that the inherent nature of C_2_ in the ground state is experimentally inaccessible. Here, we present the chemical synthesis of C_2_ from a hypervalent alkynyl-λ^3^-iodane in a flask at room temperature or below, providing experimental evidence to support theoretical predictions that C_2_ has a singlet biradical character with a quadruple bond, thus settling a long-standing controversy between experimental and theoretical chemists, and that C_2_ serves as a molecular element in the bottom-up chemical synthesis of nanocarbons such as graphite, carbon nanotubes, and C_60_.

## Abstract (web summary)

Diatomic carbon (C_2_) is historically an elusive chemical species, considered to require high physical energy for its generation. Here, the authors describe the first room-temperature chemical synthesis of C_2_ and present experimental evidence for its singlet biradical (quadruple bonding) character and role as a molecular element of nanocarbons.

## Introduction

**Fig. 1:**
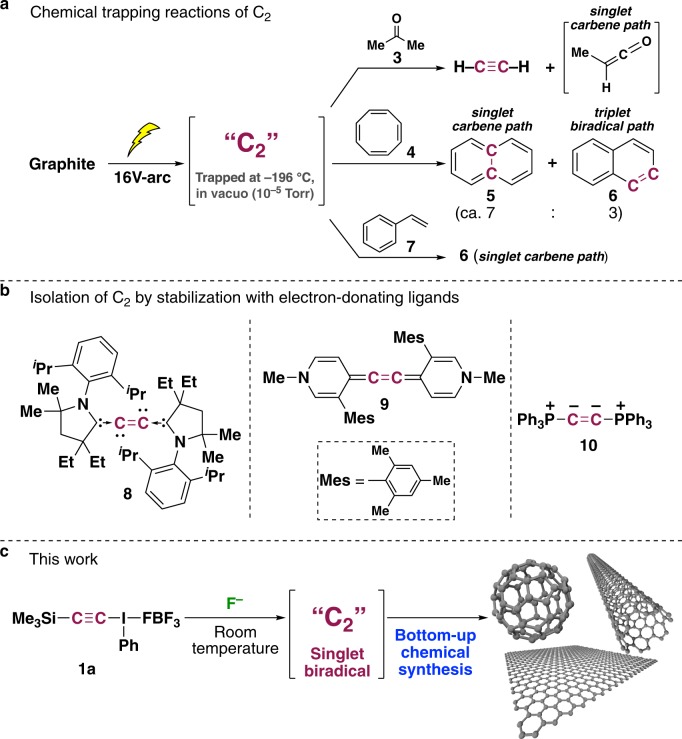
Previous experimental work on C_2_ and our synthesis of C_2_ at low temperature in a flask. **a** Chemical trapping of C_2_ generated by a carbon arc. **b** Isolation of C_2_ stabilized by potent electron-donating ligands. **c** Our chemical synthesis of C_2_ at ambient temperature under normal pressure by utilizing hypervalent alkynyl-λ^3^-iodane **1a**.

Diatomic carbon (C_2_) exists in carbon vapor, comets, the stellar atmosphere, and interstellar matter, but although it was discovered in 1857^1^, it has proven frustratingly difficult to characterize, since C_2_ gas occurs only at extremely high temperatures (above 3500 °C)^2^. Considerable efforts have been made to generate/capture C_2_ experimentally and to measure its physicochemical properties. The first successful example of artificial generation of C_2_, which was confirmed spectroscopically, involved the use of an electric carbon arc under high vacuum conditions^3^. Subsequent chemical trapping studies pioneered by Skell indicated that C_2_ behaves as a mixture of singlet dicarbene and triplet biradical states in a ratio of 7:3 to 8:2 (Fig. 1a)^4–7^. Multiple photon dissociation of two-carbon small molecules (acetylene, ethylene, tetrabromoethylene, etc.) by infrared or UV irradiation in the gas phase was also developed to generate C_2_, but this photo-generated C_2_ also exhibited several electronic states^8^. Recently, other approaches for the isolation of C_2_ have been reported, using potent electron-donating ligands to stabilize C_2_ by means of dative interactions (L:→C_2_←:L), but such stabilized complexes no longer retain the original character of C_2_ (Fig. 1b)^9–12^. Instead, theoretical/computational simulation has been applied recently, and the results indicated that C_2_ has a quadruple bond with a singlet biradical character in the ground state^13,14^.

These various theoretical and experimental findings have sparked extensive debate on the molecular bond order and electronic state of C_2_ in the scientific literature, probably because of the lack of a method for the synthesis of ground-state C_2_. Here, we present a straightforward room-temperature/pressure synthesis of C_2_ in a flask. We show that C_2_ generated under these conditions behaves exclusively as a singlet biradical with quadruple bonding, as predicted by theory. We also show that spontaneous, solvent-free reaction of in situ generated C_2_ under an argon atmosphere results in the formation of graphite, carbon nanotubes (CNTs), and fullerene (C_60_) at room temperature. This not only represents a bottom-up chemical synthesis of nanocarbons at ordinary temperature and pressure, but it also provides experimental evidence that C_2_ may serve as a key intermediate in the formation of various carbon allotropes (Fig. 1c).

## Results

### Chemical synthesis of C_2_

The key strategy underlying the present achievement is the use of hypervalent iodane chemistry^15–17^, aiming to utilize the phenyl-λ^3^-iodanyl moiety as a hyper-leaving group (ca. 10^6^ times greater leaving ability than triflate (–OSO_2_CF_3_), a so-called super-leaving group)^18^. We designed [*β*-(trimethylsilyl)ethynyl](phenyl)-λ^3^-iodane **1a**^19^, in the expectation that it would generate C_2_ upon desilylation of **1a** with fluoride ion to form anionic ethynyl-λ^3^-iodane **11**, followed by facile reductive elimination of iodobenzene. Gratifyingly, exposure of **1a** to 1.2 equivalents of tetra-*n*-butylammonium fluoride (Bu_4_NF) in dichloromethane resulted in smooth decomposition at −30 °C with the formation of acetylene and iodobenzene, indicating the generation of C_2_! However, all attempts to capture C_2_ with a range of ketones and olefins, such as acetone (**3**), 1,3,5,7-cyclooctatetraene (**4**), styrene (**7**), and 1,3,5-cycloheptatriene, failed, though they smoothly reacted with arc-generated C_2_ on an argon matrix at −196 °C^3–5,20^. These findings immediately suggested that the putative C_2_ synthesized here at −30 °C has a significantly different character from C_2_ generated under high-energy conditions (Supplementary Fig. 1).

### Experimental evidence for singlet biradical character

**Fig. 2:**
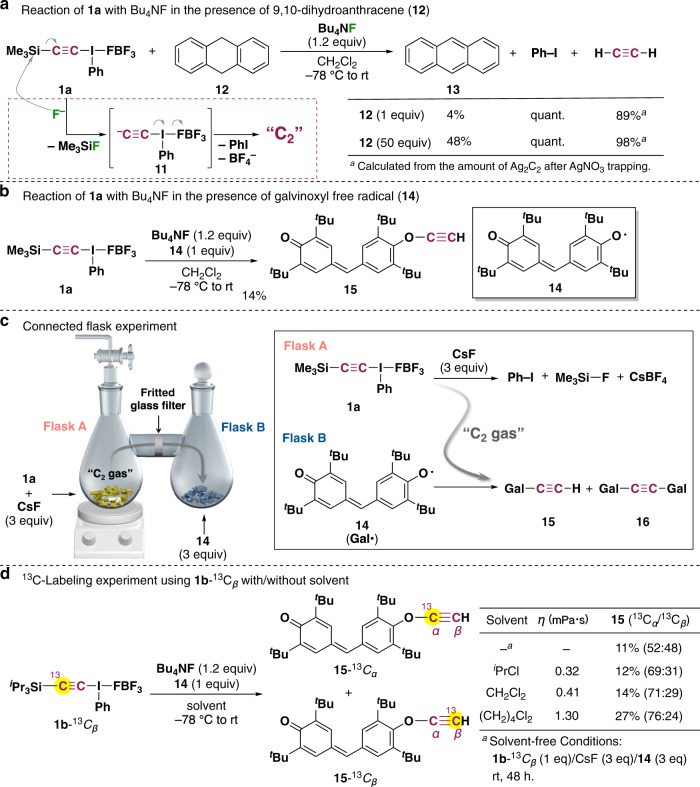
Chemical trapping of C_2_ synthesized at low temperature. **a** Reaction of **1a** with Bu_4_NF in the presence of 9,10-dihydroanthracene (**12**). **b** Reaction of **1a** with Bu_4_NF in the presence of galvinoxyl free radical **14**. **c** Connected-flask experiment. **d** ^13^C-Labeling experiments using **1b**-^13^C_*β*_.

Taking account of the fact that quantum chemical calculations suggest a relatively stable singlet biradical C_2_ with quadruple bonding in the ground state, we next examined an excellent hydrogen donor. 9,10-Dihydroanthracene has very weak C–H bonds (bond dissociation energy of **12**: 76.3 kcal mol^−1^ vs CH_2_Cl_2_: 97.3 kcal mol^−1^)^21,22^ that might effectively trap the putative singlet biradical C_2_. When **12** was added to the reaction mixture, anthracene (**13**) was obtained accompanied with the formation of acetylene (Fig. 2a), which clearly suggests that the generation of C_2_ and subsequent hydrogen abstraction from **12** gave acetylene. The formation of acetylene was confirmed by Raman spectroscopy after AgNO_3_ trapping, and the amount of acetylene was estimated by the quantitative analysis of Ag_2_C_2_ thus generated (Supplementary Fig. 2). These results strongly support the relatively stable (singlet) biradical nature of our C_2_, in accordance with the theoretical calculations. Thus, we turned our attention to the galvinoxyl free (stable) radical **14** in order to trap C_2_ directly. To our delight, *O*-ethynyl ether **15** was obtained in 14% yield, accompanied with the formation of acetylene (84%) (Fig. 2b). The structure of **15** was fully characterized by ^1^H/^13^C NMR spectra: an upfield-shifted acetylenic proton was seen at 1.78 ppm in the ^1^H NMR, as well as considerably separated ^13^C NMR chemical shifts of two acetylenic carbons (C_*α*_: 90.4 ppm and C_*β*_: 30.0 ppm), clearly indicating the presence of an ethynyl ether unit (e.g., ethynyl ethyl ether, C_*α*_: *δ* 88.2 ppm; C_*β*_: *δ* 22.0 ppm)^23^. In solution, di-galvinoxyl alkyne **16** was undetectable or barely detectable even when excess amounts of **14** were used, though **15** was obtained as almost the sole product in all cases. On the other hand, when we performed the trapping reaction in the presence of two equivalents of **14** under solvent-free conditions, **16** was clearly observed by atmospheric pressure chemical ionization (APCI) mass spectrometry (MS), although in very small quantity (Supplementary Fig. 3)^24^. These findings are consistent with the valence bond model of a singlet biradical species, according to which the energy barrier of the second hydrogen abstraction is lower by approximately 10 kcal/mol compared with the first hydrogen abstraction, which has to overcome the bonding energy of the singlet biradical^25,26^. It should be noted that the *O*-phenylated product was not formed at all, excluding alternative single electron transfer pathways, such as those via ethynyl(phenyl)-λ^2^-iodanyl radical (Supplementary Fig. 4)^27^.

In order to obtain more direct information about the generation of C_2_ “gas,” we designed a connected-flask, solvent-free experiment (Fig. 2c): a solvent-free chemical synthesis of C_2_ using **1a** with three equivalents of CsF was carried out in one of a pair of connected flasks (Flask A), and three equivalents of **14** was placed in the other flask (Flask B). The reaction mixture in Flask A was vigorously stirred at room temperature for 72 h under argon. As the reaction proceeds in Flask A, generated C_2_ gas should pass from Flask A to Flask B. Indeed, the color of **14** in Flask B gradually changed from deep purple to deep brown as the reaction progressed. After 72 h, the formation of **15** and **16** was confirmed by APCI–MS analysis of the residue in Flask B. We then performed a ^13^C-labeling experiment using **1b**-^13^*C*_*β*_, which was synthesized from H_3_^13^*C*–I in eight steps^28,29^. Treatment of **1b**-^13^*C*_*β*_ (99% ^13^C) with Bu_4_NF in the presence of **14** in CH_2_Cl_2_ gave a mixture of **15**-^13^*C*_*α*_ and **15**-^13^*C*_*β*_, suggesting that C_2_ is generated before the *O*-ethynyl bond-forming reaction with **14** (Fig. 2d). The observed *O*–^13^C/^12^C selectivity (71:29) may be related to very fast radical pairing between C_2_ and **14** prior to ejection of iodobenzene from the solvent cage^30^. We also carried out ^13^C-labeling experiments using **1b**-^13^*C*_*β*_ in solvents of different viscosities (η). The observed *O*–^13^C/^12^C selectivity decreased as the viscosity decreased, and the regioselectivity was almost lost (52:48) under solvent-free conditions. Similarly, the *O*–^13^C/^12^C selectivity was 51:49 in the connected-flask experiment. All these findings rule out stepwise addition/elimination mechanisms (Supplementary Fig. 5).

### Role as molecular element of nanocarbons

**Fig. 3:**
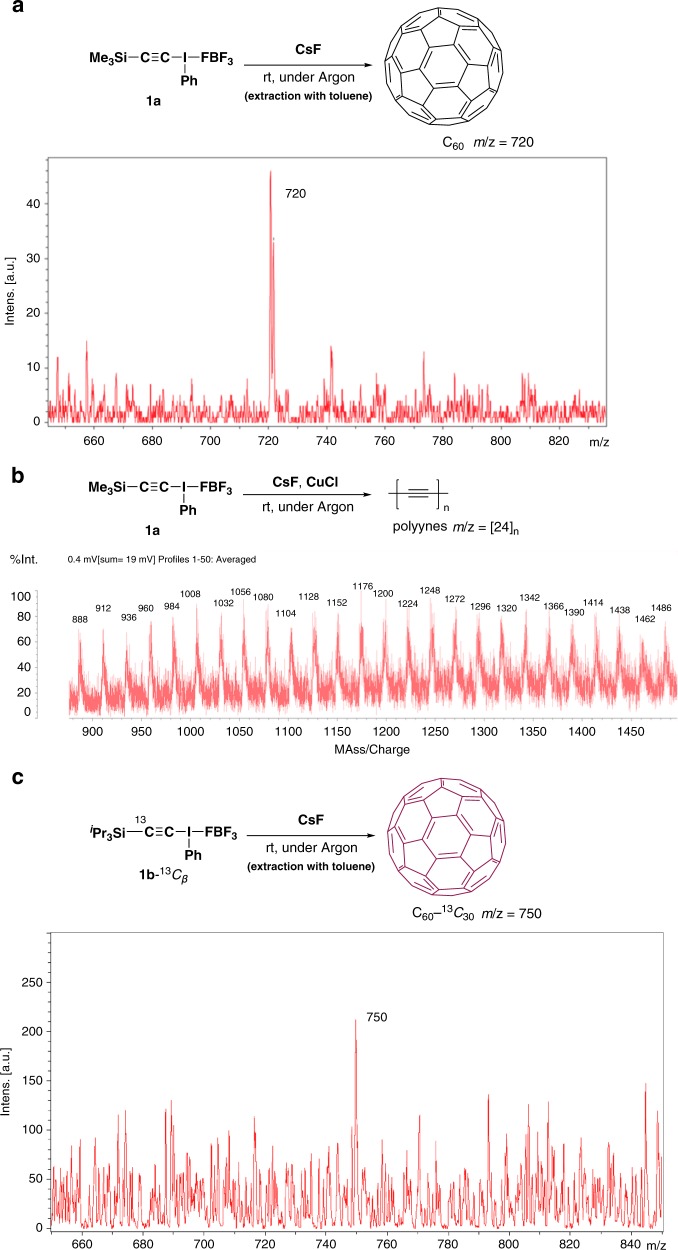
Solvent-free reaction of in situ generated C_2_ in a mortar at room temperature leads to spontaneous formation of carbon allotropes. MALDI-TOF mass spectra of **a** Ground **1a** and CsF. **b** Ground **1a** and CsF in the presence of CuCl (1.0 equiv). **c** Ground **1b**-^13^*C*_*β*_ and CsF.

**Fig. 4:**
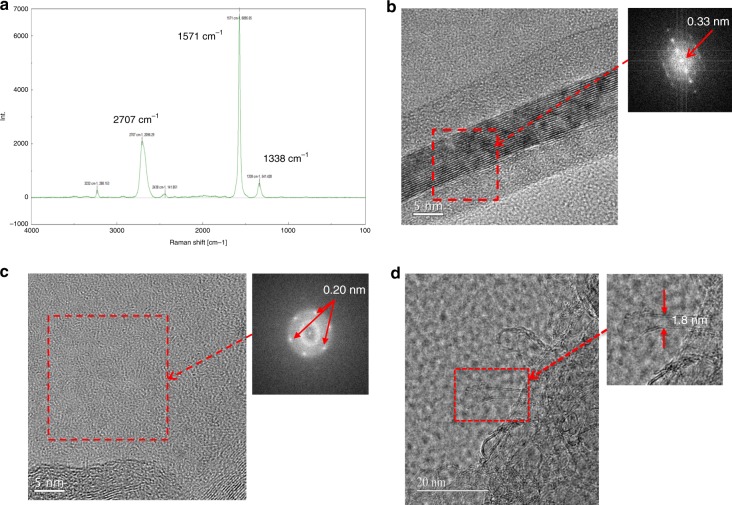
Raman spectra and HRTEM images (and their power spectra) of carbon allotropes. **a** Raman spectrum of graphite-containing sample. **b** HRTEM image and power spectrum of graphite-containing sample corresponding to the (002) lattice plane. **c** HRTEM image and power spectrum of graphite-containing sample corresponding to the (100) lattice plane. **d** HRTEM image of carbon nanotube-containing sample.

Given that C_2_ generated at room temperature or below behaves exclusively as a singlet biradical, as theoretically predicted for the ground state, we examined whether this ground-state C_2_ would serve as a molecular element for the formation of various carbon allotropes. Today, nanocarbons such as graphene, CNTs, and fullerenes, in which *sp*^2^ carbon takes the form of a planar sheet, tube, ellipsoid, or hollow sphere, are at the heart of nanotechnology^31^. But, in contrast with the rapid growth of their practical applications, the mechanisms of their formation remain unclear. Various models and theories for the growth of carbon allotropes have been proposed, most of which include the addition/insertion of C_2_ into a growing carbon cluster as a key step^32–39^. However, this idea lacks experimental verification. To investigate this issue, we examined the solvent-free reaction of the present singlet biradical C_2_ in order to avoid hydrogen quenching. Notably, simple grinding of CsF and 1.5 equivalents of **1a** in a mortar and pestle at ambient temperature for 10 min under an argon atmosphere resulted in the formation of a dark-brown solid containing various carbon allotropes, as determined by resonance Raman spectroscopy (Supplementary Fig. 6), matrix-assisted laser desorption ionization time-of-flight (MALDI-TOF) MS (Fig. 3a) and electrospray ionization (ESI) MS (Supplementary Fig. 8). Careful examination of the Raman spectra and high-resolution transmission electron micrograph (HRTEM) images indicated that high-quality graphite with few defects and an interlayer distance of 0.33 nm (Fig. 4a–c) and amorphous carbon (ca. 80–30% yields) had been mostly synthesized (Supplementary Fig. 6a), together with very small amounts of C_60_ (Fig. 3a and Supplementary Figs. 8, 9a, and 10–12) and CNTs/carboncones (Fig. 4d and Supplementary Figs. 6b, and 7)^40,41^. The chemical synthesis of double/triple-walled CNTs/carboncones has never previously been reported. We did not observe any peaks attributable to larger fullerenes, such as C_70_, C_76_, C_78_, and C_84_. This specificity may reflect the ambient temperature/pressure condition, as the electric carbon arc method generally affords a fearsome mixture of carbon allotropes.

By using **1b**-^13^*C*_*β*_, we further confirmed that C_60_ is synthesized from C_2_. Grinding of **1b**-^13^*C*_*β*_ with CsF under the same reaction conditions as above afforded C_60_-^13^*C*_30_, which was detected by means of MALDI-TOF and ESI MS, while nonlabeled C_60_ was not detected at all (Fig. 4c and Supplementary Fig. 9b). The formation of this unique fullerene is solid evidence for the role of C_2_, as its occurrence probability in nature is extremely small [(0.01)^30^]. When CuCl was added to the reaction mixture (which can stabilize alkynyl radical termini), a mixture of various fragments of polyynes –[C≡C]_*n*_– with different chain lengths was observed by MALDI-TOF MS (Fig. 4b). Such peaks were not observed from authentic C_60_, graphite, and SWCNTs (<ca. 7 nm in diameter) under the same measurement conditions. Lagow et al. proposed that linear acetylenic carbon biradicals (•[C≡C]_*n*_•) are a key intermediate/precursor for the formation of C_60_, and our results seem to support this view^34,39^.

In conclusion, we have generated C_2_ at ordinary temperature and pressure. Further, we have established that it has a singlet biradical character at low temperature, settling a long-standing difference of opinion between experimental and theoretical chemists. We also observed spontaneous formation of carbon allotropes such as graphite, CNTs, carboncones, amorphous carbon, and C_60_ from C_2_ at ambient temperature, providing the first experimental support for the generally held belief that the formation mechanism of nanocarbons involves the addition/insertion of C_2_ into a growing carbon cluster as a key step. This is also represents the first chemical synthesis of nanocarbons at ordinary temperature and pressure from C_2_ in the ground state. Easy synthetic access to in situ generated C_2_ should be helpful in opening up additional areas of chemistry and materials science, including further studies on the hot topic of the growth mechanisms of bottom-up synthesis of nanocarbons from C_2_.

## Methods

### General procedure for solid-state reaction of alkynyl-λ^3^-iodane 1a with CsF

Alkynyl-λ^3^-iodane **1a** (71 mg, 0.15 mmol) and cesium fluoride (15 mg, 0.10 mmol) were gently mixed in an agate mortar under argon, and the mixture was ground for 10 min. The color of the reaction mixture gradually changed from yellowish white to dark brown during the grinding process. The solid residue was treated with excess *t-*BuOK in order to remove remaining **1a**, and then carefully extracted with toluene (ca. 1 mL × 3) and the combined organic phase was analyzed by MALDI-TOF (Fig. 4a and Supplementary Fig. 12) and ESI MS (Supplementary Figs. 8 and 9a). LC-UV analysis was performed with TSKgel ODS-120T (250 × 4.6 mm) using toluene/MeCN = 50/50 as an eluent (Supplementary Fig. 11). LC-MS analysis was performed with Shim-Pack GIST-HP C18 (150 × 2.1 mm) using toluene/MeCN = 60/40 as an eluent (Supplementary Fig. 9). Yield of fullerene C_60_ was determined to be 4.0 × 10^−5^% by using an external standard method. As shown below, a linear calibration curve was obtained for toluene solutions of C_60_ over the concentration range from 1 to 60 ppb (1, 3, 6, 10, 30, and 60 ppb); the correlation coefficient was *R*^2^ = 0.99 (Supplementary Fig. 18).

In a separate experiment, after toluene extraction, the reaction mixture was washed several times with water, dispersed in a small amount of ethanol, and dried on a stainless steel plate, which was analyzed by Raman spectroscopy (Supplementary Fig. 6a). Further oxidative treatment in order to remove amorphous carbon from the reaction mixture was carried out as described below.

### Oxidative treatment with hydrogen peroxide

To the reaction mixture (8.0 mg) obtained above experiment was added an excess of 20% H_2_O_2_ aqueous solution (ca. 8 mL), and the mixture was heated at 100 °C for 24 h^42^. After cooling, the mixture was centrifuged (4000 rpm, 10 min) and the supernatant was removed. The residue was washed several times with deionized water and then analyzed by Raman spectroscopy (Fig. 4a and Supplementary Fig. 6b) and HRTEM (Fig. 4b, c).

### Oxidative treatment with nitric acid

To the reaction mixture (48 mg) obtained above experiment was added an excess of 3.2 M HNO_3_ aqueous solution (ca. 48 mL) and the mixture was heated at 100 °C for 24 h^43^. After cooling, the mixture was filtered and washed with deionized water, followed by four times with 4 M NaOH aqueous solution and finally with deionized water. The residue was then analyzed by Raman spectroscopy and HRTEM (Fig. 4d and Supplementary Fig. 7).

### Experimental data

For experimental procedures and spectroscopic data of the compounds, see Supplementary Information. For general procedures for alkynyl-λ^3^-iodanes **1b**-^13^*C*_*β*_ and trapping reactions, see Supplementary Methods and Supplementary Figs. 1 and 2. For Raman, HRTEM, ESI mass, LC-ESI mass, UV–Vis, LC-UV chromatograms, and MALDI-TOF mass spectra of a sample obtained by a solvent-free reaction, see Supplementary Figs. 6–12. For NMR spectra see Supplementary Figs. 13–17.

## Supplementary information

Supplementary Information

## Data Availability

Detailed experimental procedures and characterization of compounds can be found in the Supplementary Information (Supplementary Figs. 1–18 and Supplementary Methods). All data are available from the authors on reasonable request.
