# Supplementary material for: Room-temperature chemical synthesis of C2
Source: Nat Commun. 2020 May 1;11:2134. doi: 10.1038/s41467-020-16025-x (PMC7195449; doi:10.1038/s41467-020-16025-x)
Supplement: Supplementary file 1 — Supplementary Information [file 41467_2020_16025_MOESM1_ESM.pdf]

Supplementary Information

Room-temperature Chemical Synthesis of C_2

Miyamoto et al.

Supplementary Methods

General

All air-sensitive manipulations were carried out in an argon-filled glovebox or by standard Schlenk techniques under argon. NMR spectra were obtained on a Bruker Ascend™ 500 spectrometer. NMR spectra were processed with Topspin 3.5 (Bruker). Chemical shifts are expressed in δ (ppm) values. ^{1}H and ^{13}C NMR spectra were referenced to tetramethylsilane (0 ppm) and $CHCl_3$ (7.26, 77.4 ppm) as internal standards. Coupling constants are reported in hertz. IR spectra were obtained on a JASCO FT/IR-4700 spectrometer. Raman spectra were obtained on either a NRS-4500 or NRS-5500 spectrometer. Matrix-assisted laser desorption ionization time-of-flight (MALDI-TOF) mass spectra were recorded either on a Bruker-Daltonics microflex LT or a Shimadzu AXIMA® ResonanceTM in the linear mode. Electrospray ionization (ESI) mass spectra and atmospheric pressure chemical ionization (APCI) mass spectra were recorded on a Bruker micrOTOF-II spectrometer. LC-ESI-MS spectrometric analyses were performed on a Shimadzu LC-2040C Plus and a Shimadzu LCMS-2020 using a Shimadzu Shim-pack GIST-HP column (C18, 2.1 × 150 mm, 3 μm), which was maintained at 40˚C. The temperature of the auto-sampler was set at 15˚C. High-resolution transmission electron microscopy (HRTEM) was done on a JEOL JEM-2100F microscope (200 kV). High-resolution transmission electron microscopy (HRTEM) was done on a JEOL JEM-2100F microscope (200 kV). The electronic absorption spectra were recorded with a JASCO V-670 spectrophotometer. Normal-phase column chromatographic separation was performed with silica gel 60 (230–400 mesh) from Merck. Thin-layer chromatography was carried out on 0.25 mm Merck silica gel plates (60F-254).

Solvents and Reagents

Olefins (1,3,5,7-cyclooctatetraene (**4**), styrene (**7**)), 9,10-dihydroanthracene (**12**), galvinoxyl free radical (**14**), bis(trimethylsilyl)acetylene, (diacetoxyiodo)benzene, trimethylsilyl triflate, boron trifluoride acetonitrile complex, triisopropylsilyl chloride, trimethyltin chloride, iodomethane-^{13}*C*, triethyl phosphite, lithium diisopropylamide, methyl acetate, sodium hydride, tosyl azide, 4-methoxybenzyl chloride, ethylene glycol, *tetra*-n-butylammonium iodide, Dess-Martin reagent,

2,3-dichloro-5,6-dicyanobenzo-*p*-quinone (DDQ), manganese(IV) oxide, cesium fluoride, potassium fluoride, fullerene, carbon nanotubes (<ca. 7 nm), graphite, and [2-(trimethylsilyl)ethynyl](phenyl)(tetrafluoroborato)-λ^3-iodane (**1a**)[1] were purchased from Aldrich Inc., FUJIFILM Wako Pure Chemical Co., Tokyo Kasei Co., and other commercial suppliers, and used as received. Acetone (**3**), dichloromethane (DCM), 2-chloropropane, 1,4-dichlorobutane, and tetrahydrofuran (THF) were purchased from Kanto Chemical Co., Inc., degassed by purging with argon, and/or dried with a solvent purification system containing a one-meter column of activated alumina.

2-1. Chemical synthesis of alkynyl-λ^3-iodanes 1b-$^{13}C_\beta$

(1) ^{13}C-Labeled Ohira-Bestmann reagent **17** was prepared according to the reported procedure (eq. 1).[2]

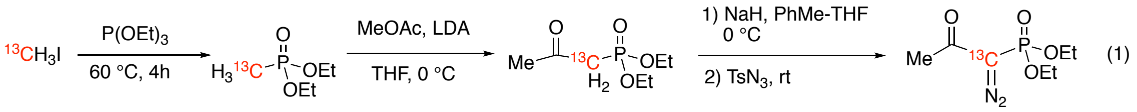

17

(2) 2-(4-Methoxybenzyloxy)acetaldehyde (**18**) was prepared according to the reported procedure (eq. 2).[3]

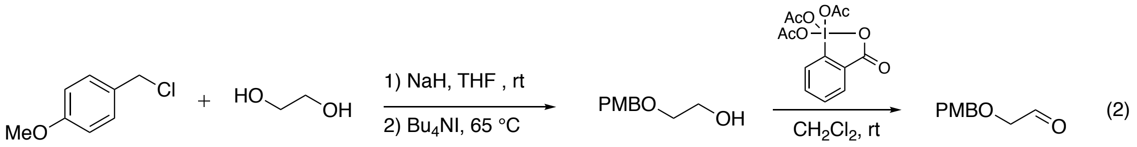

PMB: 4-methoxybenzyl

18

(3) 3-[4-(Methoxybenzyl)]prop-1-yne-^{13}C (**19**) was prepared according to the reported procedure (eq. 3).[2]

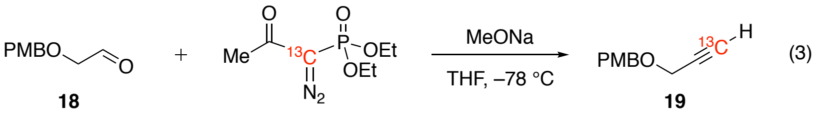

18 **19**

(4) 1-(Triisopropylsilyl)acetylene-^{13}C (**20**)[3] was prepared according to the reported procedure (eq. 4).[4]

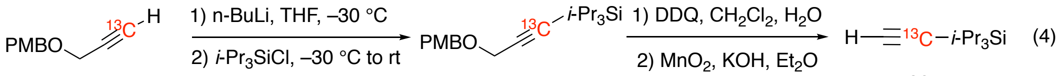

19 **20**

(5) Trimethyl[2-(triisopropylsilyl)ethynyl]stannane-^{13}C (**21**) was prepared according to the reported procedure (eq. 5).[5]

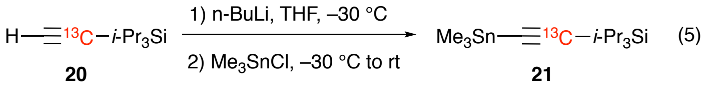

20 **21**

(6) Synthesis of alkynyl-λ^3-iodanes **1b-**$^{13}C_\beta$ from **21**.

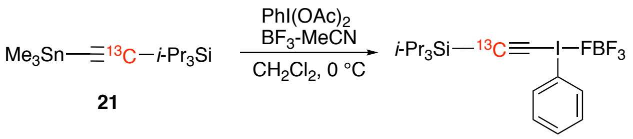

21

1b–$^{13}C_\beta$

To a stirred solution of (diacetoxyiodo)benzene (174 mg, 0.54 mmol) in dichloromethane (10 mL) was added BF_3-MeCN (486 μL, 0.59 mmol) and trimethyl[2-(triisopropylsilyl)ethynyl]stannane-^{13}C (**21**) (205 mg, 0.59 mmol) at 0 °C

under argon. The mixture was stirred at the temperature for 1 h, and then poured into 46% aqueous HBF_4 solution at 0 °C. The organic phase was collected and washed twice with 46% aqueous HBF_4 solution. The aqueous phase was extracted with dichloromethane four times. The combined organic phase was filtered and concentrated under reduced pressure to give a yellow oil, which was washed with hexane several times. The oil was further purified by recrystallization from dichloromethane-hexane at –30 °C to give pure **1b**-$^{13}C_{\beta}$ (180 mg, 70%) as a white solid. ATR-FTIR (neat) (cm^{-1}) 2944, 2866, 1466, 1446, 1210–890, 982, 917, 881, 718, 673. ^{1}H NMR (500 MHz, $CDCl_3$) δ 8.08 (d, J = 8.4 Hz, 2H), 7.69 (t, J = 7.4 Hz, 1H), 7.57 (dd, J = 8.4, 7.4 Hz, 2H), 1.06–1.18 (m, 21H, TIPS). ^{13}C NMR (125 MHz, $CDCl_3$) δ 133.5, 133.03, 132.95, 121.1, 116.0, 38.7, 18.3, 11.1, 11.0.

Trapping Reaction of alkynyl-λ^{3}-iodane 1a with Bu_4NF in the presence of 9,10-dihydroanthracene 12 (Fig. 3a)

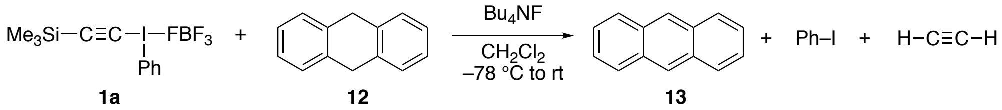

To a stirred solution of alkynyl-λ^{3}-iodane **1a** (19.4 mg, 0.05 mmol) and 9,10-dihydroanthracene (**12**) (450 mg, 2.5 mmol) in dichloromethane (1.5 mL) was slowly added a solution of tetra-n-butylammonium fluoride trihydrate (18.9 mg, 0.06 mmol) in dichloromethane (1 mL) at –78 °C under argon. The mixture was gradually warmed to room temperature over 3 h, and then water (3 mL) was added. The organic phase was separated and the aqueous phase was extracted with dichloromethane four times. The combined organic phase was dried over anhydrous sodium sulfate and concentrated under reduced pressure to give a yellow solid. ^{1}H NMR analysis (reference: 1,1,2,2-tetrachloroethane) showed the formation of anthracene (**13**) (48%) and iodobenzene (100%). The residue was purified by column chromatography (hexane) to give **13** (2.1 mg, 12%) as a white solid.[6] ^{1}H NMR (500 MHz, $CDCl_3$) δ 8.44 (s, 2H), 8.02–8.00 (m, 4H), 7.46–7.44 (m, 4H). The amount of acetylene was determined by trapping experiments using silver nitrate. In a separate experiment, the same reaction was carried out using **1a** (50.5 mg, 0.112 mmol) in one of a pair of connected flasks (vide infra), and silver nitrate (95.2 mg, 0.560 mmol) in water (5.5 ml) was placed in the other flask prior to addition of tetra-n-butylammonium fluoride. The

reaction mixture was warmed to room temperature, and the white precipitate formed in the silver nitrate solution was collected by vacuum filtration, and washed several times with deionized water and then with MeOH to give silver acetylide (22.0 mg, 85%) as a white solid. CAUTION: silver acetylide in the dry state readily explodes upon mechanical shock, and only a small quantity should be handled.

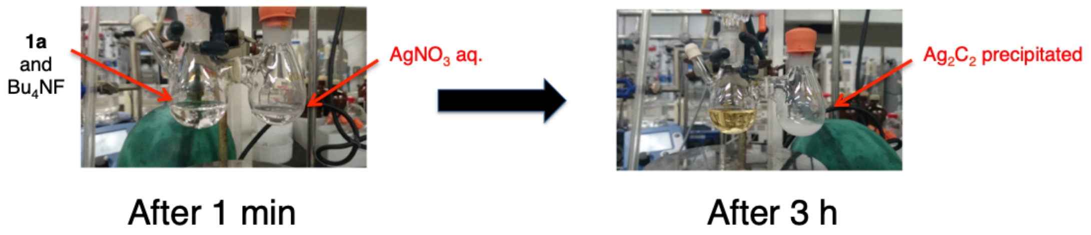

Although the Raman shift of silver acetylide varies depending upon the conditions, the Raman shift of the white solid obtained was in good agreement with that of an authentic sample prepared from acetylene gas and aqueous silver nitrate: Raman (ν): 1856 cm^{-1} (see also Supplementary Fig. 2a).[7] The formation of acetylene in the gas phase of the reaction flasks was also directly confirmed by IR and Raman spectroscopies. Raman (ν): 1974 cm^{-1} (see also Supplementary Figs. 2c and 2e).[8]

Trapping reaction of alkynyl-λ^3-iodane 1a with Bu_4NF in the presence of galvinoxyl free radical 14 (Fig. 3b)

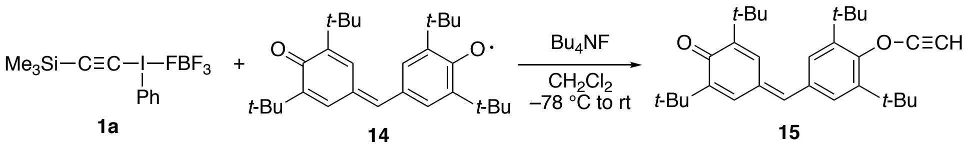

To a stirred solution of alkynyl-λ^3-iodane **1a** (50 mg, 0.13 mmol) and galvinoxyl free radical **14** (54 mg, 0.13 mmol) in dichloromethane (2 mL) was slowly added a solution of *tetra*-n-butylammonium fluoride trihydrate (47 mg, 0.15 mmol) in dichloromethane (2 mL) at –78 °C under argon. The mixture was gradually warmed to room temperature over 3 h, and then concentrated under reduced pressure to give a purple oil, which was purified by preparative TLC (hexane:ethyl acetate = 14:1 using a PLC plate pre-developed with dichloromethane:triethylamine = 5 : 1) to give **15** (8.1 mg, 14%) as a yellow oil. ATR-FTIR (neat) (cm^{-1}) 2957, 2179, 2149, 1613, 1360, 1187, 1117, 914, 883, 734. ^1H NMR (500 MHz, $CDCl_3$) δ 7.50 (d, J = 2.2 Hz, 1H), 7.41 (s, 2H), 7.15 (s, 1H), 7.01 (d, J = 2.2 Hz, 1H), 1.78 (s, 1H, C≡CH), 1.40–1.25 (m, 36H). ^{13}C NMR (125

MHz, $CDCl_3$) δ 186.5, 155.3, 149.6, 147.8, 143.3, 142.1, 135.1, 133.5, 131.9, 130.2, 129.1, 127.6, 90.4 (*C*≡CH), 36.2, 35.5, 35.0, 31.5, 30.6, 30.3, 30.0 (C≡*C*H), 29.6, 29.5, 29.4. HRMS (APCI (+)) *m/z* calcd for $C_{31}H_{43}O_2$ [$(M+H)^+$] 446.3185, found 446.3187.

Solvent-free trapping reaction in the presence of galvinoxyl free radical 14 (two-pot experiment, Fig. 3c)

1a (30.5 mg, 0.08 mmol) and cesium fluoride (35.8 mg, 0.24 mmol) was placed in one of a pair of connected flasks (Flask A), and galvinoxyl **14** (100 mg, 0.24 mmol) was placed in the other flask (Flask B). The reaction mixture in Flask A was vigorously stirred at room temperature for 72 hours under argon. The contents of Flask B was directly analyzed by APCI mass spectrometry. $^{1}H/^{13}C$ NMR analysis was conducted after exposure of the reaction mixture to excess 1,4-cyclohexadiene in order to quench remaining **14**.

^{13}C-Labeling experiment using 1b-$^{13}C_\beta$ as a trapping agent (Fig. 3d)

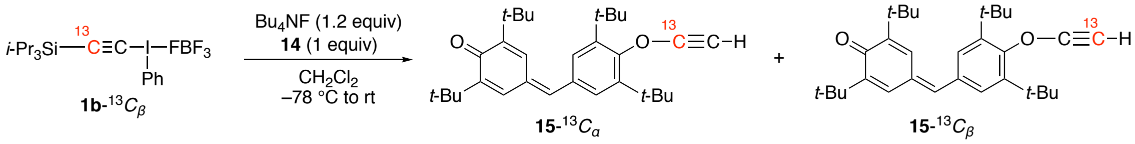

To a stirred solution of alkynyl-λ^3-iodane **1b**-$^{13}C_\beta$ (28 mg, 0.06 mmol) and galvinoxyl free radical **14** (25 mg, 0.06 mmol) in dichloromethane (1 mL) was slowly added a solution of *tetra*-n-butylammonium fluoride (23 mg, 0.07 mmol) in dichloromethane (1 mL) at –78 °C under argon. The mixture was gradually warmed to room temperature over 3 h, and then concentrated under reduced pressure to give a purple oil, which was purified by preparative TLC (hexane:ethyl acetate = 14:1 using a PLC plate pre-developed with dichloromethane:triethylamine = 5 : 1) to give a 7:3 mixture of **15**-$^{13}C_\alpha$ and **15**-$^{13}C_\beta$ (3.8 mg, 14%) as a yellow oil. The ratio of **15**-$^{13}C_\alpha$ and **15**-$^{13}C_\beta$ was determined by ^{1}H and ^{13}C NMR analysis. ATR-FTIR (neat) (cm^{-1}) 2956, 2116, 1613, 1361, 1187, 1114, 911, 884, 732. ^{1}H NMR (500 MHz, $CDCl_3$) δ 7.50 (d, J = 2.2 Hz, 1H), 7.41 (s, 2H), 7.15 (s, 1H), 7.01 (d, J = 2.2 Hz, 1H), 1.78 (d, $^2J_{CH}$ = 59.7 Hz, 0.7H for **15**-$^{13}C_\alpha$; $^1J_{CH}$ = 264.9 Hz, 0.3H for **15**-$^{13}C_\beta$), 1.40–1.25 (m, 36H). ^{13}C NMR (125 MHz, $CDCl_3$) δ 186.5, 155.3, 149.6, 147.8, 143.3, 142.1, 135.1, 133.5, 131.9, 130.2, 129.1, 127.6, 90.4 (*C*≡CH), 36.2, 35.5, 35.0, 31.5, 30.6, 30.3, 30.0 (C≡*C*H), 29.6, 29.5, 29.4.

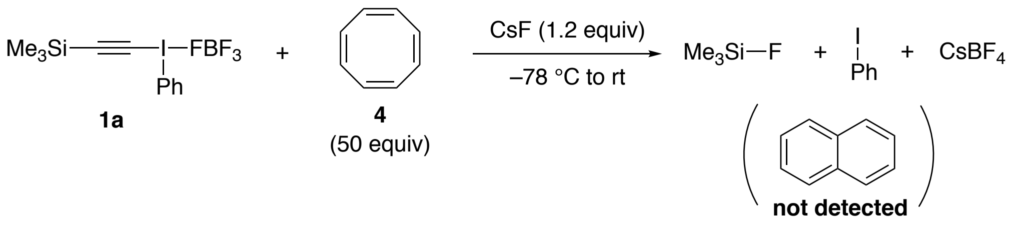

Supplementary Fig. 1 Reaction of 1a with CsF in the presence of 4 under solvent-free conditions.

All attempts to capture C_2 with a range of ketones and olefins such as acetone (**3**), 1,3,5,7-cyclooctatetraene (**4**), styrene (**7**), and 1,3,5-cycloheptatriene were unsuccessful.

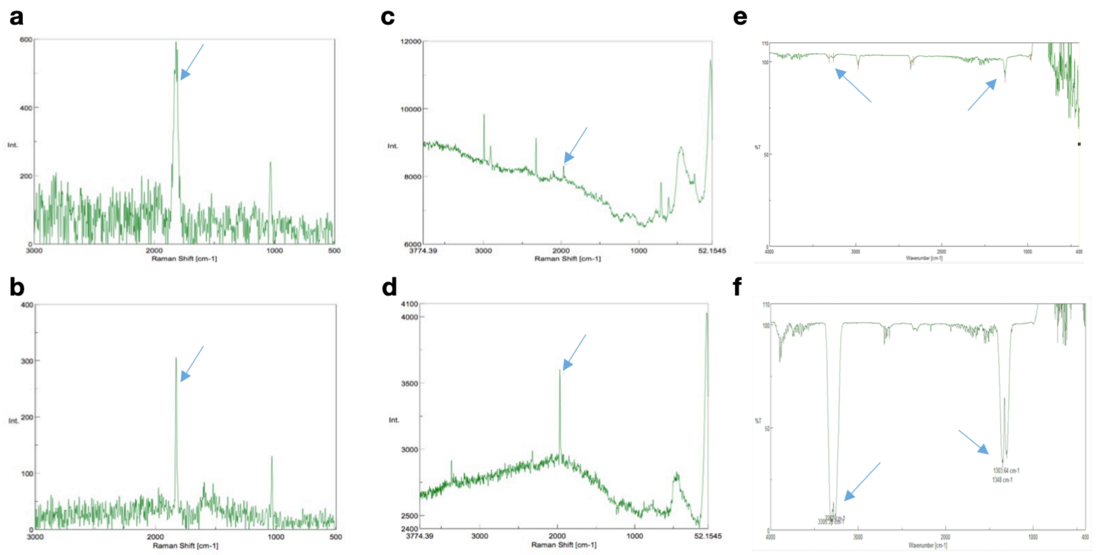

Supplementary Fig. 2 Raman spectra (a–d) and IR spectra (e and f) of acetylene and silver acetylide. a, Silver acetylide obtained from 1a–Bu_4NF mixture and $AgNO_3$. b, Authentic sample of silver acetylide. c, Acetylene obtained from 1a–Bu_4NF mixture. d, Authentic sample of acetylene. e, Acetylene obtained from 1a–CsF mixture. f, Authentic sample of acetylene.

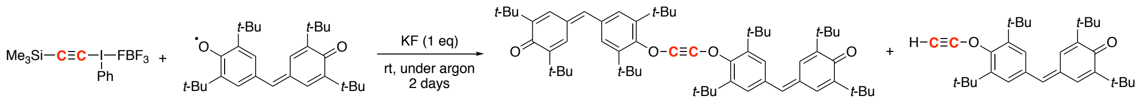

1a · **14** *m/z* 421 (2 eq) · **16** *m/z* 866 major · **15** *m/z* 446 minor

The formation of **16** was clearly observed by APCI mass spectrometry, but because of its highly labile character, all attempts to isolate **16** were unsuccessful. ^{1}H NMR yield of **16** was estimated to be <5% after exposure of the reaction mixture with excess 1,4-cyclohexadiene.

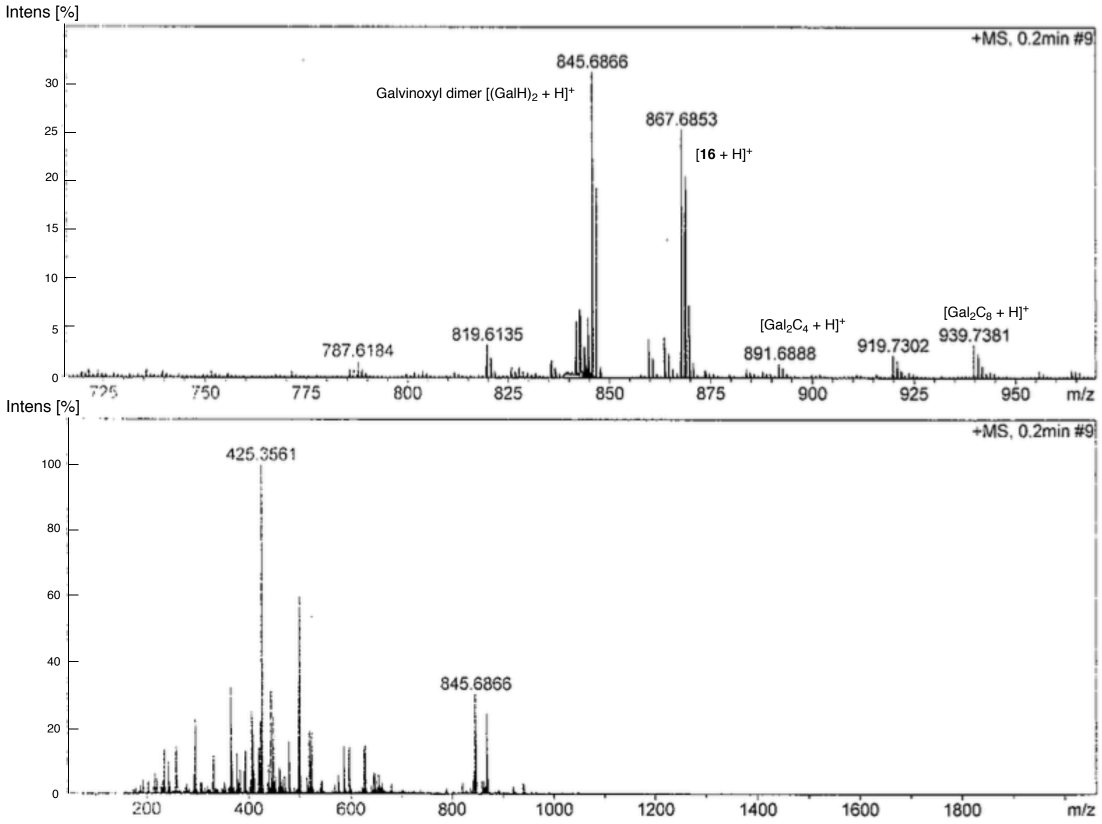

Supplementary Fig. 3 APCI mass spectrum (positive ion mode) of the reaction mixture of a trapping experiment using 2 equivalents of 14 under solvent-free conditions.

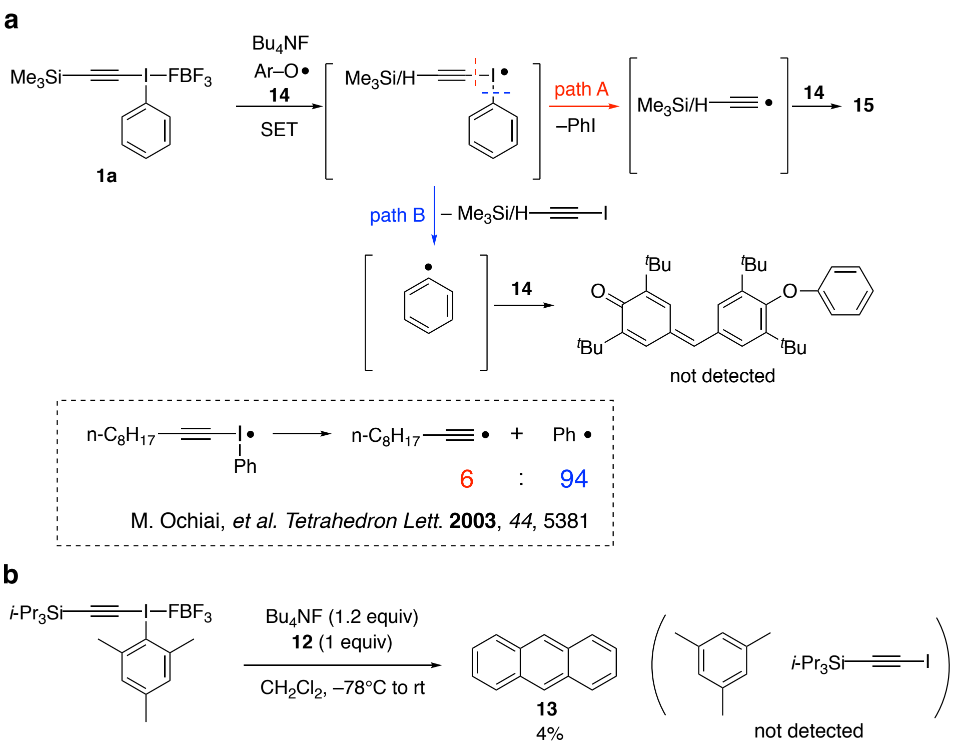

Supplementary Fig. 4 a, Plausible SET pathway for the formation of 15. b, Control experiment excluding the SET pathway.

a

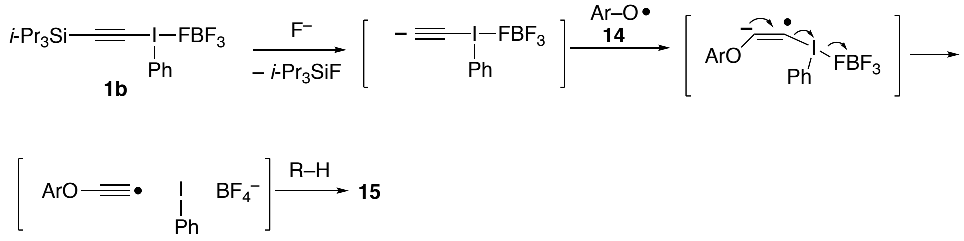

b

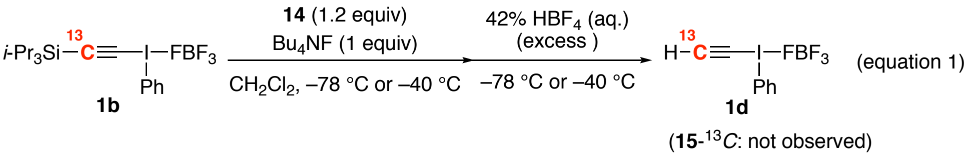

c

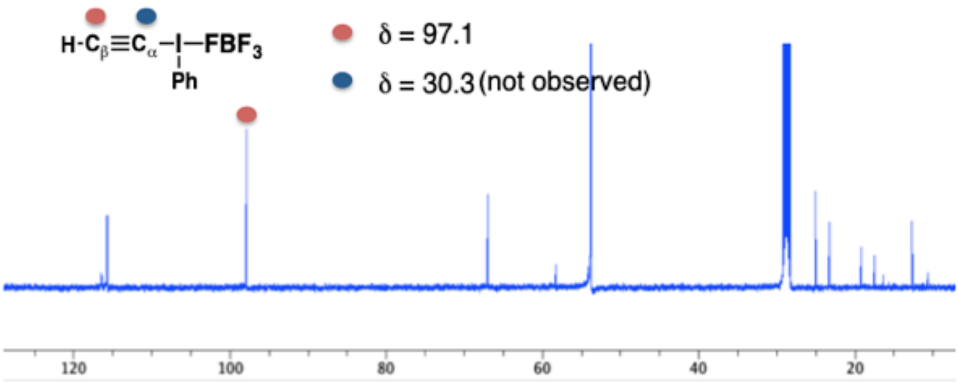

Supplementary Fig. 5 a, Plausible stepwise addition/elimination mechanisms for the formation of 15. b, Control experiment with 1b-$^{13}C_\beta$. c, ^{13}C NMR spectrum of 1d-$^{13}C_\beta$ in the reaction mixture (125 MHz, acetone-d_6).

In order to rule out this pathway, we performed the reaction of **1b**-$^{13}C_\beta$ with Bu_4NF in the presence of 1.2 equivalents of **14** in dichloromethane at –78 ºC or –40 °C, followed by quenching with 42% aq. HBF_4. This reaction afforded ethynyl-λ^3-iodane **1d**-$^{13}C_\beta$ as the sole product. Neither the regioisomeric **1d**-$^{13}C_\alpha$ (*i.e.*, there is no ^{13}C-scrambling) nor the galvinoxyl adducts such as **15** or **16** were observed in the reaction.

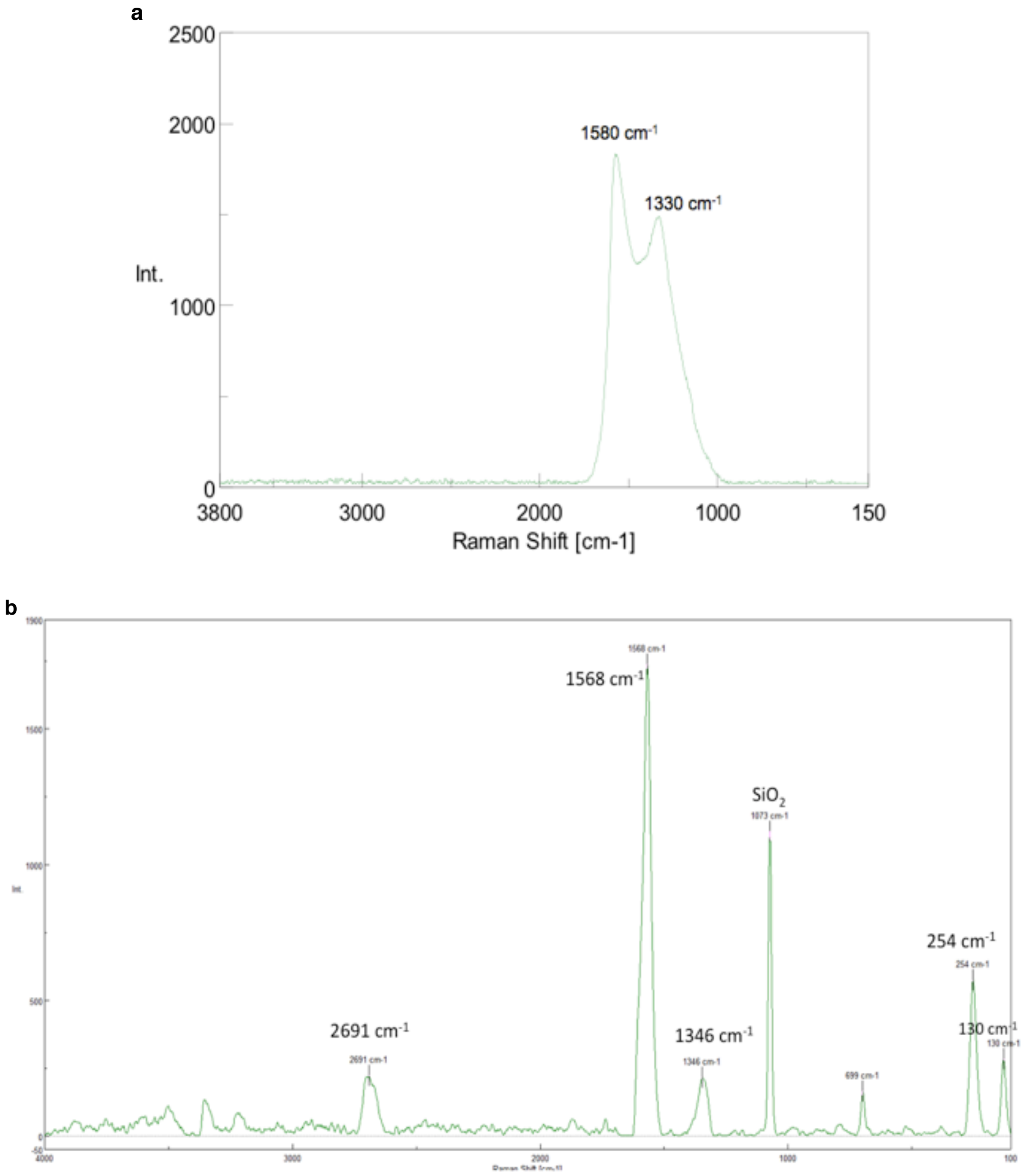

Supplementary Fig. 6 Raman spectra of a sample obtained by a solvent-free reaction. a, Before oxidation treatment. b, After oxidation treatment (20% H_2O_2 aqueous solution at 100 °C for 24 h).[9]

For spectrum a, the G and D band peaks appearing at 1580 cm^{-1} and 1330 cm^{-1} were similar to those of diamond-like carbon with high C_{sp}^{3} content (ta-C).[10] For spectrum b, the peak appearing at 130 cm^{-1} might suggest the formation of CNT with *ca.* 2 nm diameter (see Fig. 5d).[11]

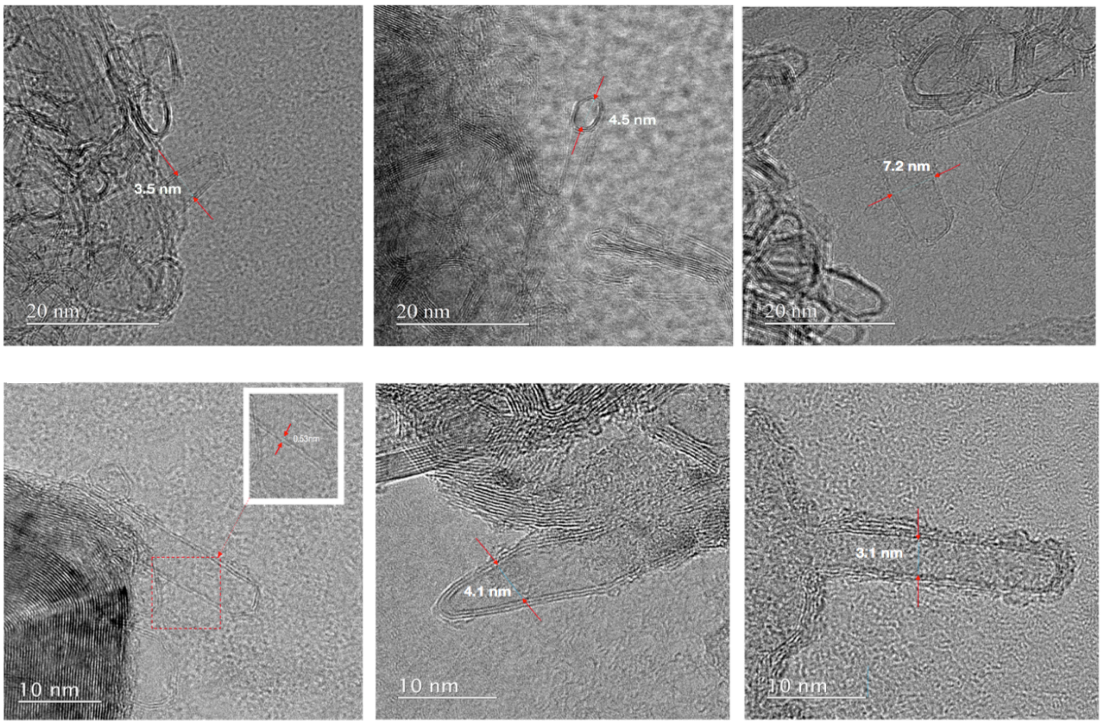

Supplementary Fig. 7 HRTEM images of a carbon nanotube/carboncone-containing sample obtained by a solvent-free reaction after oxidative work-up (3.2 M aqueous HNO_3, 100 °C, 24 h).[12]

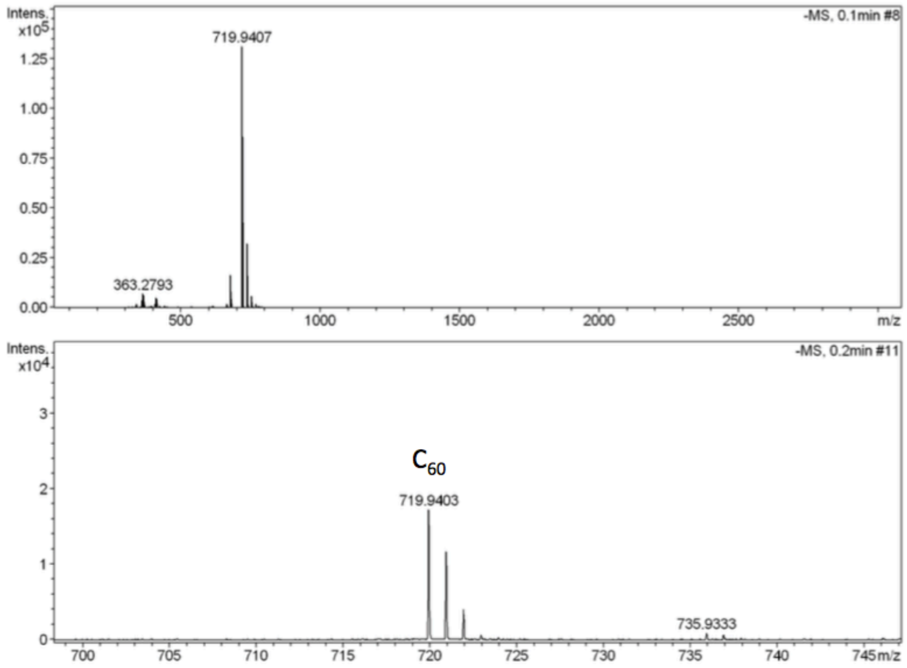

Supplementary Fig. 8 ESI mass spectrum (negative ion mode) of a toluene extract obtained by a solvent free reaction.

We did not get a clear-cut ion peaks attributed to C_{70} (*m/z* 840), C_{76} (*m/z* 912), C_{78} (*m/z* 936), or C_{84} (*m/z* 1008). Small ion peak of *m/z* 736 can be attributed to C_{60}-oxide ($C_{60}O$).[13]

a

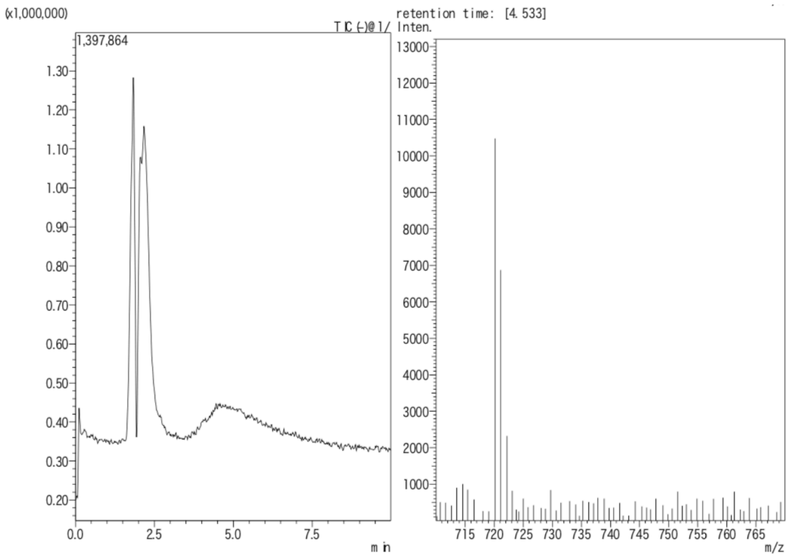

b

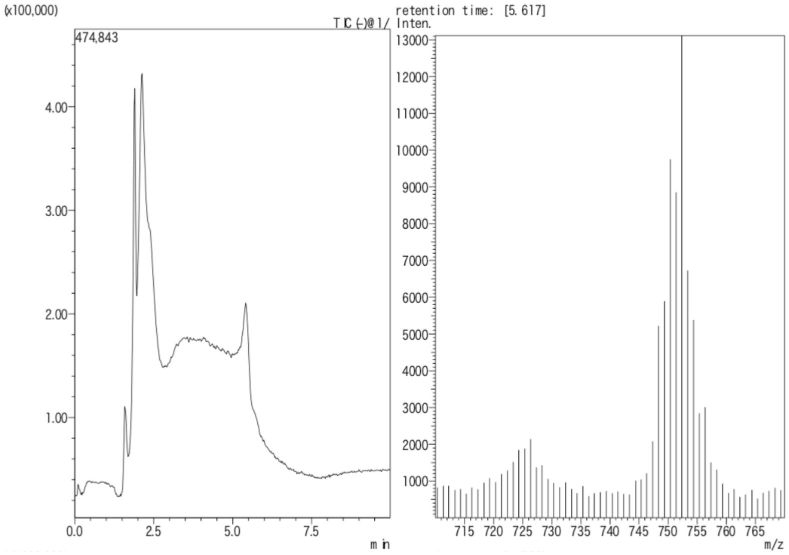

Supplementary Fig. 9 LC-ESI mass chromatograms (negative ion mode) and spectra of C_{60} and C_{60}-$^{13}C_{30}$. a, C_{60}-containing sample obtained by a solvent-free reaction. A total ion current chromatogram (left column) and mass spectrum observed at the retention time of 4.5 min (right column). b, C_{60}-$^{13}C_{30}$ containing sample obtained by a solvent-free reaction (using 1b-$^{13}C_{\beta}$). A total ion current chromatogram (left column) and mass spectrum observed at the retention time of 5.6 min (right column).

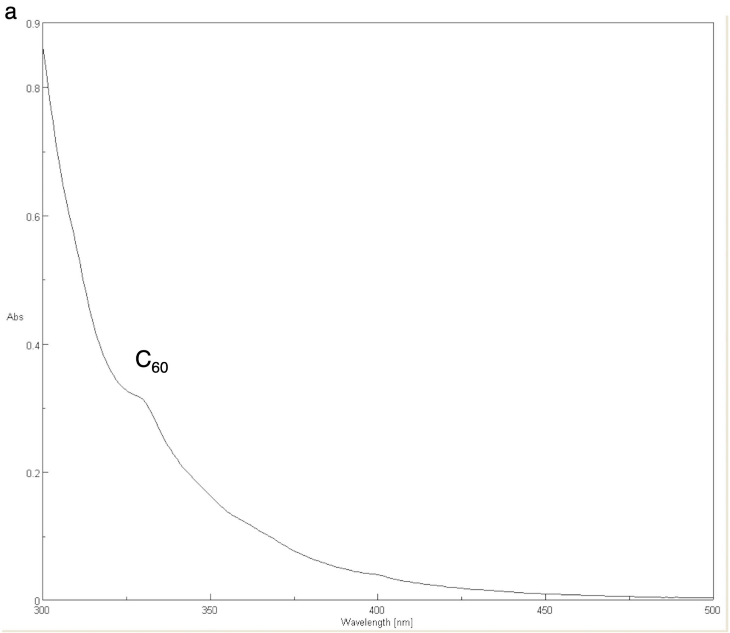

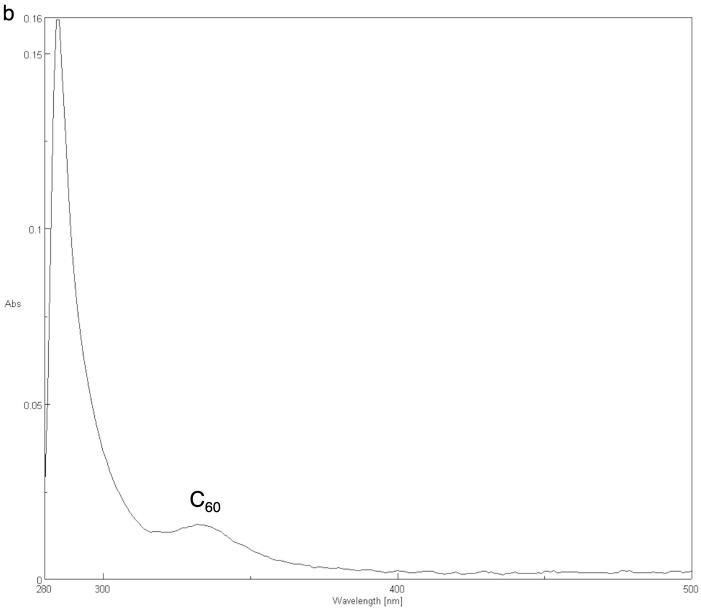

Supplementary Fig. 10 UV-Vis spectra of a, A toluene extract obtained by a solvent free reaction. b, Authentic sample of fullerene (1 ppb).

The absorption band observed in the λ = 330–340 nm region can be assigned to the HOMO –> LUMO+1 transition of fullerene.[14]

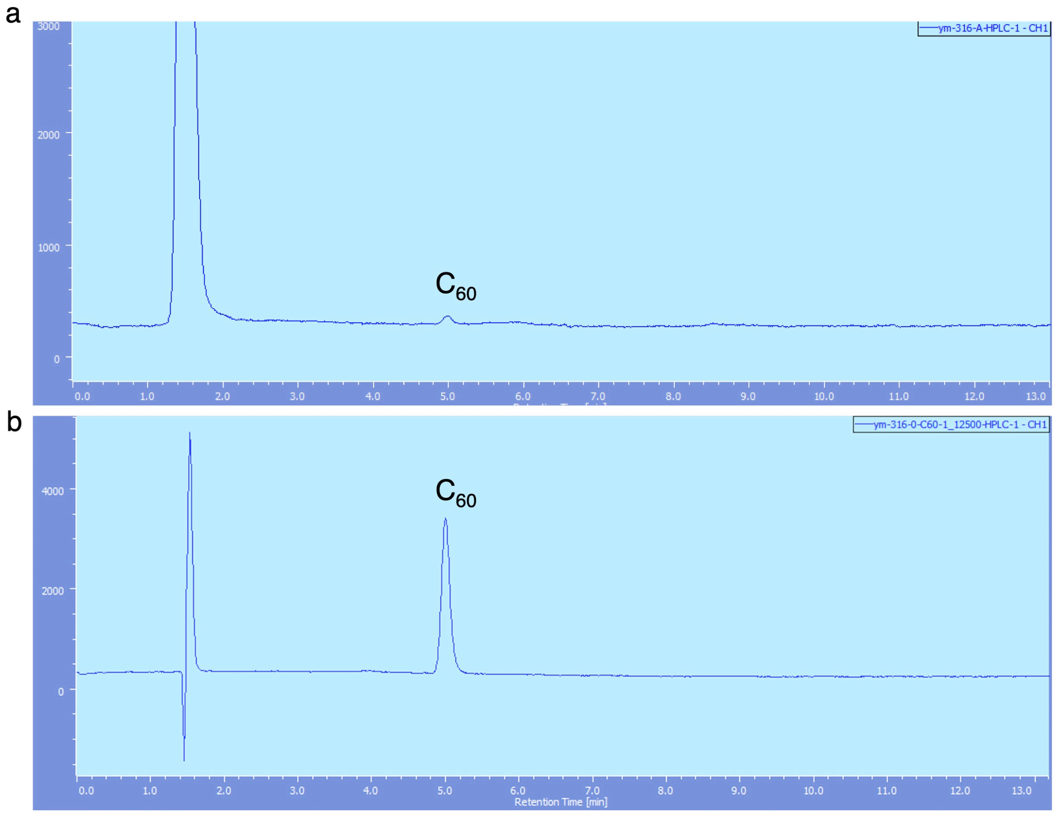

Supplementary Fig. 11 LC-UV chromatograms (254 nm) of a, A toluene extract obtained by a solvent free reaction. b, Authentic sample of C_{60}.

The weak peak observed at *ca.* 5 min was similar to that of authentic C_{60}. Column: TSKgel ODS-120T (250 x 4.6 mm); eluent: toluene/MeCN = 50/50.

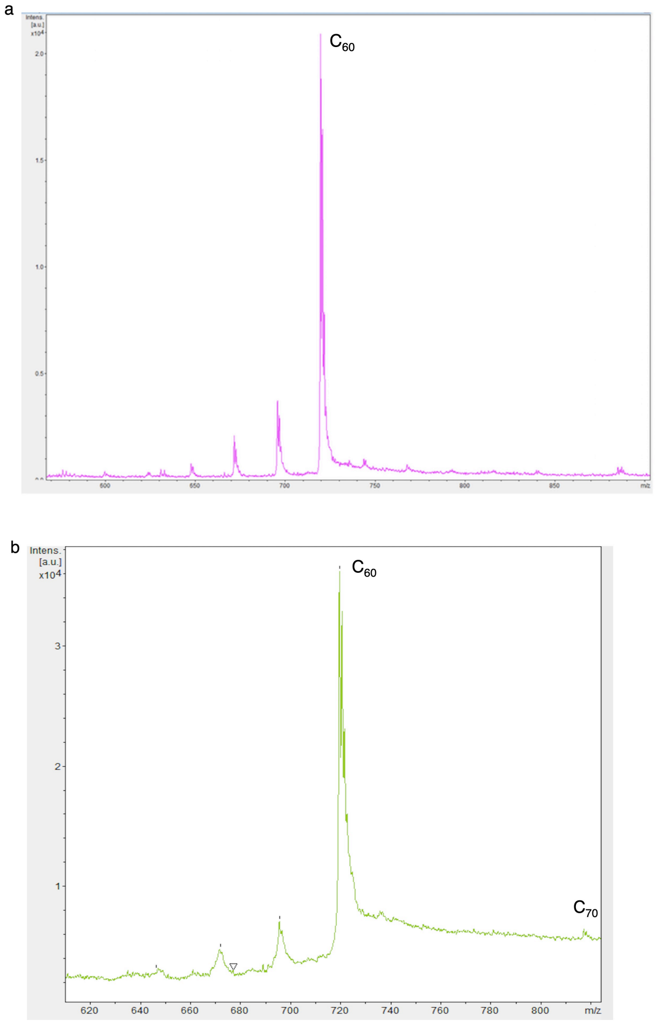

Supplementary Fig. 12 MALDI-TOF mass spectra of a, A toluene extract obtained by a solvent free reaction. b, Authentic sample of C_{60}.

A prominent ion peak at *m/z* = 720 was observed in toluene extract obtained by a solvent free reaction. In the spectrum, a peak corresponding to C_{70} (*m/z* = 840) was not detected to a discernible extent. This sample lot is different from that used for ESI mass analysis (Supplementary Fig. 8).

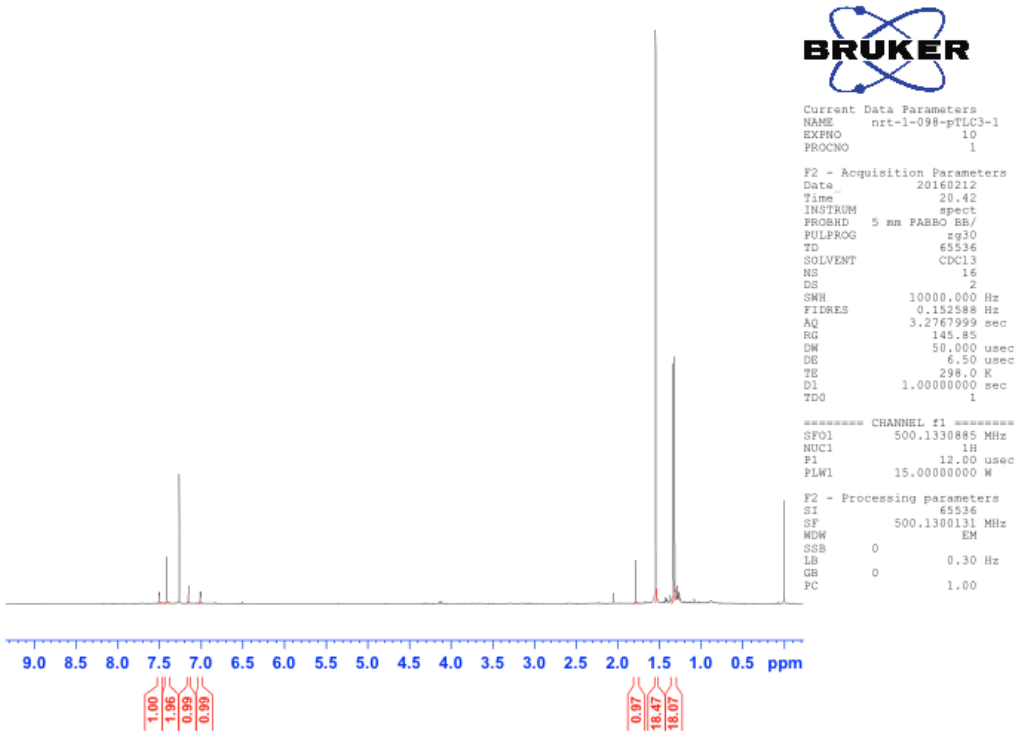

Supplementary Fig. 13 ^{1}H NMR spectra of **15**.

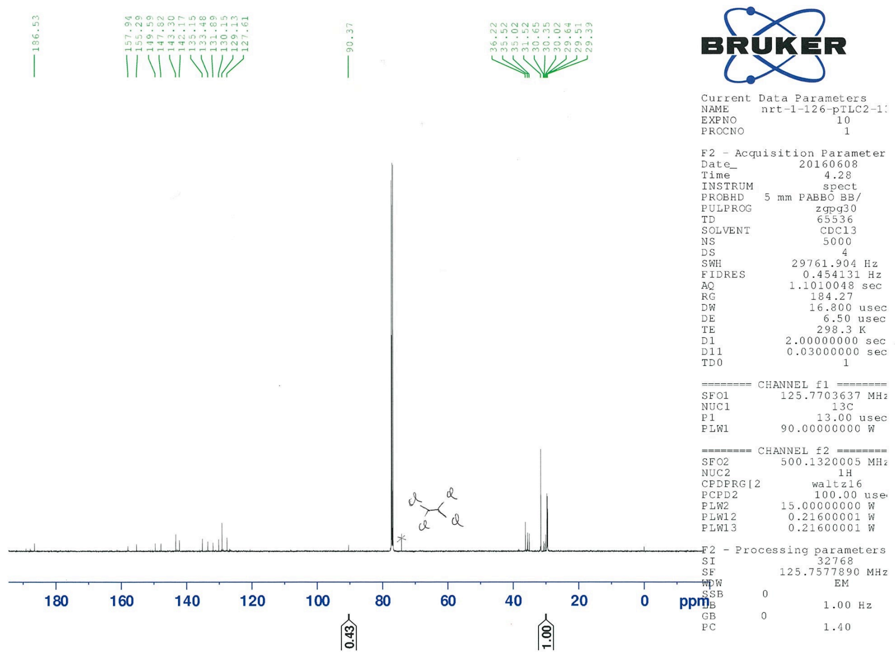

Supplementary Fig. 14 ^{13}C NMR spectra of **15**.

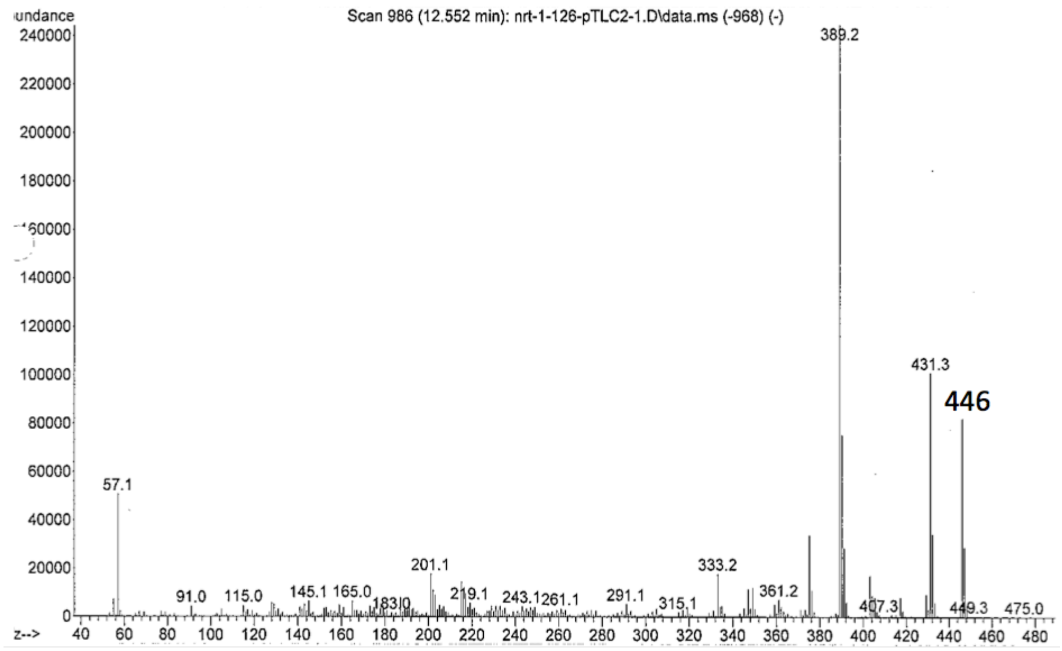

Supplementary Fig. 15 EI-MS spectrum of **15**. Three assignable peaks, *m/z* 446 for [M^+], 431 for [$(M\text{-Me})^+$] 389 for [$(M\text{-}t\text{-Bu})^+$], were observed.

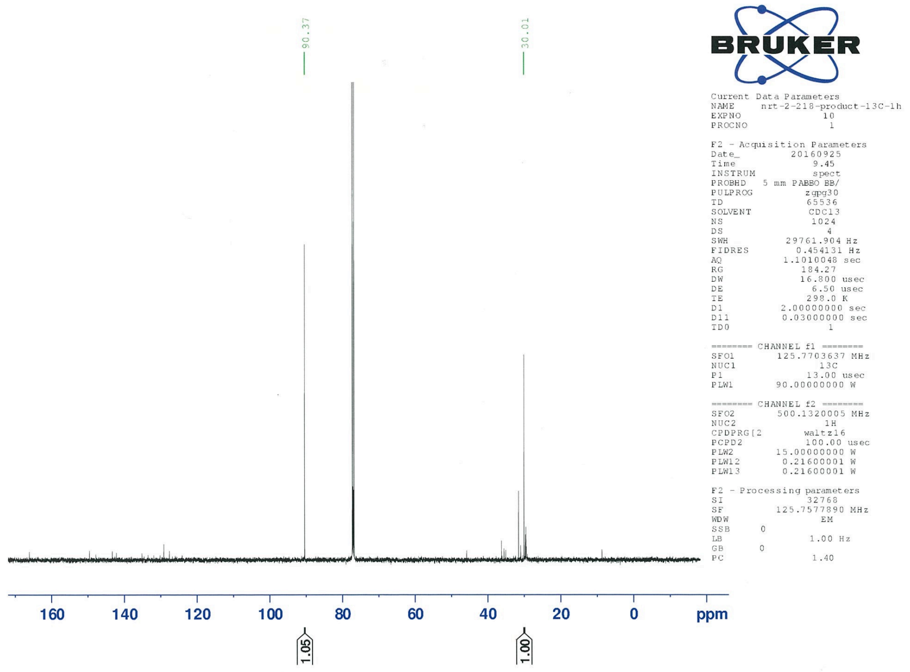

Supplementary Fig. 16 ^{13}C NMR spectrum of **15**-^{13}C in the reaction mixture.

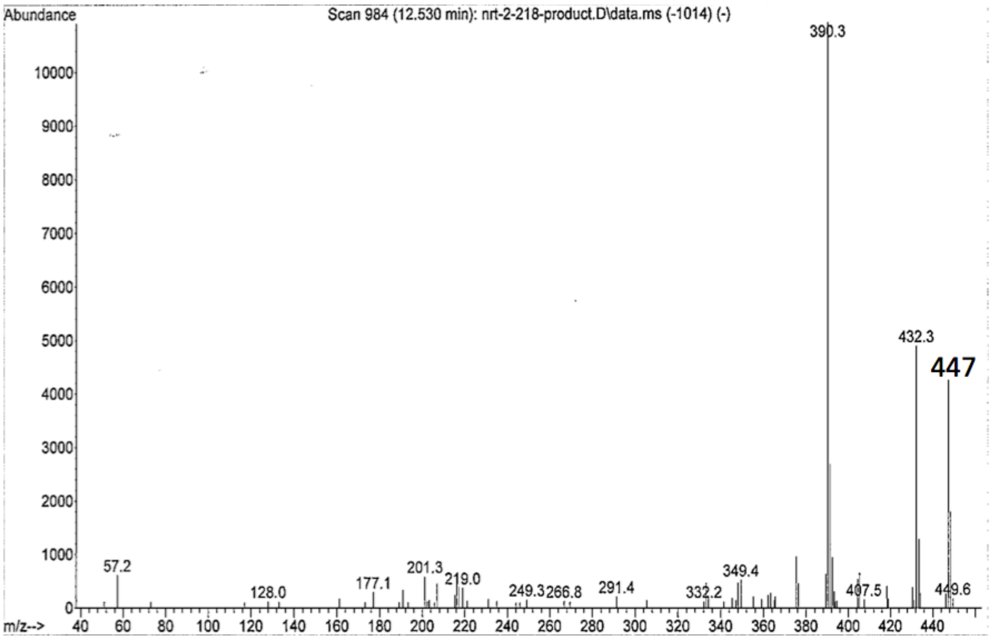

Supplementary Fig. 17 EI-MS spectrum of **15**-^{13}C. Three assignable peaks, *m/z* 447 for [M^{+}], 432 for [$(M\text{-Me})^{+}$] 390 for [$(M\text{-}t\text{-Bu})^{+}$], were observed.

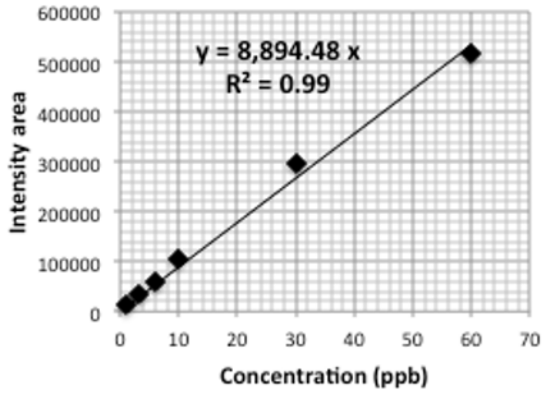

Supplementary Fig. 18 The linear calibration curve between C_{60} concentrations and MS intensity data.

Supplementary References

1 Ochiai, M., Kunishima, M., Sumi, K., Nagao, Y., Fujita, E., Arimoto, M. & Yamaguchi, H. Reaction of alkynyltrimethylsilanes with a hypervalent organoiodine compound: A new general synthesis of alkynyliodonium salts. *Tetrahedron Lett.* **26**, 4501 – 4504 (1985). Doi: 10.1016/S0040-4039(00)88941-8.

2 Brunner, A. & Hintermann, L. A sequential homologation of alkynes and aldehydes for chain elongation with optional ^{13}C-labeling. *Chem. Eur. J.* **22**, 2787–2792 (2016). Doi: 10.1002/chem.201504248.

3 Sridhar, R., Srinivas, B. & Rao, K. R. Asymmetric synthesis of triacetyl-D-erythro-sphingosine and D-1-deoxyallonojirimycin via Miyashita C2 selective endo-mode azide opening of 2,3-epoxy alcohol. *Tetrahedron* **65**, 10701–10708 (2009). Doi: 10.1016/j.tet.2009.10.035.

4 Fernández González, D., Brand, J. P. & Waser J. Ethynyl-1,2-benziodoxol-3(1*H*)-one (EBX): an exceptional reagent for the ethynylation of keto, cyano, and nitro esters. *Chem. Eur. J.* **16**, 9457–9461 (2010). Doi: 10.1002/chem.201001539.

5 Moloney, M. G., Pinhey, J. T. & Roche, E. G. The α-alk-1-ynylation of β-dicarbonyl compounds and nitronate salts by alk-1-ynyl-lead triacetates. *J. Chem. Soc., Perkin Trans.* 1 333–341 (1989). Doi: 10.1039/P19890000333.

6 Tobisu, M., Nakamura, R., Kita, Y. & Chatani, N. Rhodium-catalyzed reductive cleavage of carbon–cyano bonds with hydrosilane: A catalytic protocol for removal of cyano groups. *J. Am. Chem. Soc.* **131**, 3174–3175 (2009). Doi: 10.1021/ja810142v.

7 Guo, G.-C., Zhou, G.-D. & Mak, C. W. T. Structural variation in novel double salts of silver acetylide with silver nitrate: fully encapsulated acetylide dianion in different polyhedral silver cages. *J. Am. Chem. Soc.* **121**, 3136–3141 (1999). Doi: 10.1021/ja984117n.

8 Fast, H. & Welsh, H. L. High-resolution Raman spectra of acetylene, acetylene-d_1, and acetylene-d_2. *J. Mol. Spect.* **41**, 203–221 (1972). Doi: 10.1016/0022-2852(72)90133-6.

9 Feng, Y.; Zhang, H.; Hou, Y.; McNicholas, T. P.; Yuan, D.; Yang, S.; Ding, L.; Feng, W.; Liu, J. Room temperature purification of few-walled carbon nanotubes with high yield. *ACS Nano* **2**, 1634–1638 (2008). Doi: 10.1021/nn800388g.

10 Ferrari, A. C. & Robertson, J. Raman spectroscopy of amorphous, nanostructured, diamond-like carbon, and nanodiamond. *Phil. Trans. R. Soc. Lond.* A **362**, 2477–2512 (2004). Doi: 10.1098/rsta.2004.1452.

11 Zhao, X., Ando, Y., Qin, L.-C., Kataura, H., Maniwa, Y. & Saito, R. Radial breathing modes of multiwalled carbon nanotubes. *Chem. Phys. Lett.* **36**, 1169–1174 (2002). Doi: 10.1016/S0009-2614(02)00955-7.

12 Shao, L., Tobias, G., Salzmann, C. G., Ballesteros, B., Hong, S. Y., Crossley, A., Davis, B. G. & Greena, M. L. H. Removal of amorphous carbon for the efficient sidewall functionalisation of single-walled carbon nanotubes. *Chem. Commun.* 5090–5092 (2007). Doi: 10.1039/B712614J.

13 Deng, J.-P., Mou, C.-Y. & Han, C.-C. Electrospray and Laser Desorption Ionization Studies of C60O and Isomers of C60O2. *J. Phys. Chem.* **99**, 14907–14910 (1995). Doi: 10.1021/j100041a001.

14 Heath, J. R., Curl, R. F. & Smalley, R. E. The UV absorption spectrum of C60 (buckminsterfullerene): A narrow band at 3860Å. *J. Chem. Phys.* **87**, 4236–4238 (1987). Doi: 10.1063/1.452879.
